# Generalists or Specialists? Testing Genetic Specificity of *Leucocytozoon* Lineages and Black Fly Vectors in Thailand

**DOI:** 10.3390/biology15010028

**Published:** 2025-12-23

**Authors:** Waraporn Jumpato, Wannachai Wannasingha, Kingkan Sakundet, Chavanut Jaroenchaiwattanachote, Tongjit Thanchomnang, Wanchai Maleewong, Peter H. Adler, Pairot Pramual

**Affiliations:** 1 Department of Biology, Faculty of Science, Mahasarakham University, Kantharawichai District, Mahasarakham 44150, Thailand; waraporn.a2536@gmail.com (W.J.); kingkansakundet@gmail.com (K.S.); 2 Center of Excellence in Biodiversity Research, Mahasarakham University, Mahasarakham 44150, Thailand; wannachai.w@msu.ac.th (W.W.); chavanut.j@msu.ac.th (C.J.); 3 Faculty of Medicine, Mahasarakham University, Mahasarakham 44000, Thailand; tongjit.t@msu.ac.th; 4 Department of Parasitology, Faculty of Medicine, Khon Kaen University, Khon Kaen 40002, Thailand; wanch_ma@kku.ac.th; 5 Mekong Health Science Research Institute, Khon Kaen University, Khon Kaen 40002, Thailand; 6 Department of Plant and Environmental Sciences, Clemson University, Clemson, SC 29634, USA; padler@clemson.edu

**Keywords:** avian haemosporidians, coevolution, parasite diversity, vector–parasite specificity

## Abstract

The diversity, genetic differentiation, and cophylogeny of avian protozoa of the genus *Leucocytozoon* and their black fly vectors (Diptera: Simuliidae) in Thailand were investigated. *Leucocytozoon* infections were detected in 46 (11.2%) of 410 black flies representing six species: *Simulium asakoae*, *S. chumpornense*, *S. khelangense*, *S. siamense* complex, *S. yvonneae*, and *S. gombakense*. The latter three species represent the first reports of having *Leucocytozoon* infections, suggesting their potential role in parasite transmission. A total of 12 *Leucocytozoon* lineages was identified, including novel lineages with low sequence similarity (92%) to previously known records in the *S. siamense* complex and *S. yvonneae*. These findings indicate uncharacterized parasite diversity in wild birds of Thailand. Genetic differentiation among *Leucocytozoon* populations was minimal across different geographic populations, but highly significant among parasites in different species of black flies, even when collected from the same location. Cophylogeny analyses revealed significant co-phylogenetic relationships between *Leucocytozoon* lineages and their potential black fly vectors, despite similarities in host-blood sources. These findings emphasize that parasite–vector specificity is shaped not only by host preference, but also by coadaptation mechanisms between black flies and *Leucocytozoon* species. Further studies on avian hosts, bloodmeal sources, and parasite–vector interactions are necessary for understanding *Leucocytozoon* transmission dynamics in Southeast Asia.

## Simple Summary

Understanding host–vector–parasite relationships is essential for effective disease control. Black flies are blood-sucking insects that transmit a variety of pathogens causing diseases in humans and other animals. Avian blood parasites of the genus *Leucocytozoon* are transmitted exclusively by black flies, with only one known exception. In this study, we examined the diversity and genetic specificity between *Leucocytozoon* parasites and black fly species from Thailand. Six species of black flies were genetically screened for *Leucocytozoon* infection. In total, 12 *Leucocytozoon* lineages were detected, three of which are novel and genetically distinct from previously recorded lineages. Phylogenetic analyses revealed evidence of cospeciation between black fly species and *Leucocytozoon* lineages. Lineages infecting closely related species of black flies were genetically more similar, suggesting that coadaptation between *Leucocytozoon* parasites and their black fly vectors might be an important mechanism driving vector–parasite specificity.

## 1. Introduction

Knowledge of parasite–vector–host interactions is essential for understanding disease epidemiology [1,2,3] and for controlling associated diseases [4]. Key questions in this system are whether parasites are transmitted only by particular vector species (specialists) or by diverse vectors (generalist), and what are the mechanisms that drive such patterns. Several factors can shape parasite–vector relationships, including genetic and physiological compatibility [5], vector-feeding behavior [6], and abiotic factors such as climate [7].

*Leucocytozoon* Sambon, 1908 (Haemospororida: Plasmodiidae) is a genus of haemosporidian parasites infecting a wide range of avian hosts [8]. Morphologically, at least 45 species have been described [9], but molecular studies have revealed much greater diversity, with more than 1500 lineages recognized (1542 lineages in the MalAvi database) [10], (accessed 31 October 2024). *Leucocytozoon* infections are generally subclinical; however, some species, such as *Leucocytozoon simondi* Mathis & Léger, 1910, *L. marchouxi* Mathis & Léger, 1910, and *L. toddi* Sambon, 1908, are pathogenic. The disease leucocytozoonosis, caused by *Leucocytozoon* infections, can reduce growth rate and egg production, and in severe cases can cause host mortality. Symptoms are typically more severe in young birds than in adults and in poultry compared with wild birds [11,12].

All *Leucocytozoon* species are transmitted by black flies (Diptera: Simuliidae), except *Leucocytozoon* (*Akiba*) *caulleryi* Mathis & Léger, 1909, which is transmitted by biting midges (Diptera: Ceratopogonidae) [13,14]. At least 48 species of black flies serve as vectors of *Leucocytozoon* [14,15,16,17,18,19]. The majority of *Leucocytozoon* species can be transmitted by multiple black fly species. For example, the pathogenic species *Leucocytozoon simondi* is transmitted by at least seven species (*Cnephia ornithophilia* Davies, Peterson & Wood, 1962; *Simulium anatinum* Wood, 1963; *S. dogieli* (Rubtsov, 1956); *S. meigeni* (Rubtsov & Carlsson, 1965); *S. rugglesi* Nicholson & Mickel, 1950; *S. usovae* (Golini, 1987); and the *S. venustum* Say, 1823 complex). Similarly, *L. schoutedeni* Rodhain, Pons, Vandenbranden & Bequaert, 1913, a blood protozoan parasite infecting domestic chickens [20], is transmitted by various black fly species [17]. However, some *Leucocytozoon* lineages exhibit vector specificity, such as the IGRYS1 lineage, which has been found only in *S. annulus* (Lundström, 1911) [6].

In Thailand, the prevalence of *Leucocytozoon* infections in vertebrate hosts varies from 2–8% in wild birds and 29% in captive birds [21] up to 89% in domestic chickens [22]. At least three black fly species (*Simulium asakoae* Takaoka & Davies, 1995, *S. chumpornense* Takaoka & Kuvangkadilok, 2000, and *S. khelangense* Takaoka, Srisuka & Saeung, 2022) are candidate vectors of *Leucocytozoon* among birds, especially domestic chickens in Thailand [16,23]. Previous studies also found genetic associations between *Leucocytozoon* lineages and black fly species, particularly *S. asakoae* vs. *S. khelangense*/*S. chumpornense* [23]. Jumpato et al. [23] proposed that these *Leucocytozoon*–simuliid associations result from parasite–vector coadaptation in response to seasonal temperature, which influences the phenology of the black flies. To further investigate this hypothesis, we examined *Leucocytozoon* infections in co-occurring black fly species and tested the genetic differentiation and cophylogeny between the parasites and their vectors.

## 2. Materials and Methods

### 2.1. Collection and Identification of Black Flies

**Figure 1:**
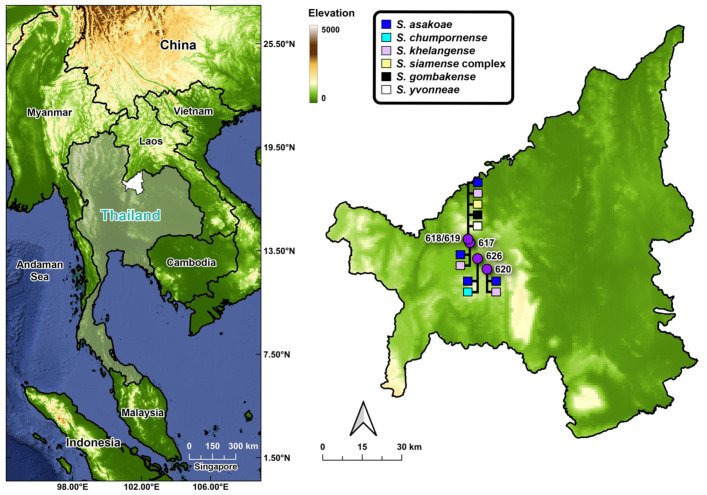
Sampling sites in Loei Province, northeastern Thailand, indicated by location codes, for adult black flies used in the molecular detection of *Leucocytozoon*. Details of each sampling site are provided in Table 1.

Five collections of black flies were made in villages, around animal pens at the forest edge, and in the forest at three sites in Phu Ruea District, Loei Province, in northeastern Thailand on 5 March 2022 and 4–5 February 2023 (Figure 1 and Table 1). Adult black flies were collected by sweeping aerial nets in a figure-8 motion 0.5–2.0 m above ground. Specimens were fixed in 80% ethanol and stored at −20 °C until processed. Black flies were morphologically identified using a key to the black flies of Thailand [24]. Because females of some closely related species (*Simulium siamense* Takaoka & Suzuki, 1984 complex and *S. yvonneae* Takaoka & Low, 2018; *S. chumpornense* and *S. khelangense*) are difficult to identify morphologically, DNA barcoding was also used.

### 2.2. DNA Barcoding of Black Flies

The GF-1 Nucleic Acid DNA extraction kit (Vivantis Technologies Sdn Bhd, Subang Jaya, Malaysia) was used to extract DNA from individuals, following the manufacturer’s protocol. The primers LCO1490 and HCO2198 [25] were used to amplify an approximately 650-bp fragment of the COI gene. The PCR reaction conditions followed those of Tangkawanit et al. [26]. The PCR products were dyed with Novel Juice (GeneDireX, Taoyuan, Taiwan, Republic of China) and examined using 1% agarose gel electrophoresis. Successful amplifications were subsequently purified using the PureDirex PCR CleanUp & Gel Extraction Kit (Bio-Helix, New Taipei City, Taiwan, Republic of China), following the manufacturer’s instructions. The purified PCR products were sequenced at ATCG Company Limited (Thailand Science Park, Pathumthani, Thailand) with the same primers used for PCR.

### 2.3. Molecular Detection of Leucocytozoon

DNA was extracted from individual black flies, using the same method as in the COI gene study. The nested PCR method and primer described by Hellgren et al. [27], with the PCR reaction conditions of Jumpato et al. [16], were used to amplify an approximately 500-bp fragment of the cyt b gene. PCR products were checked, purified, and sequenced using the same procedure as for the COI gene but with the specific primers for *Leucocytozoon*.

### 2.4. Data Analysis

COI sequences from black flies (accession nos. PX452929–PX452974) and cyt b from *Leucocytozoon* (accession nos. PX511765–PX511810) were checked for quality using the “Edit/View Sequencer Files” option in MEGA X [28]. The COI sequences (650 bp) of the black flies were compared with those in the NCBI GenBank, and conspecific sequences were retrieved for phylogenetic analyses. Phylogenetic relationships of black flies were examined using neighbor joining (NJ) and maximum likelihood (ML) methods. The NJ tree was inferred in MEGA X using the Kimura 2-parameter model and bootstrapping with 1000 replications for testing branch support. The ML tree was also inferred in MEGA X using the GTR+I+G model. Branch support was calculated using bootstrapping with 1000 replications. The output trees were visualized in FigTree v.1.4.3 (https://tree.bio.ed.ac.uk/software/figtree/) (accessed on 30 July 2025).

*Leucocytozoon* lineages were identified using the BLAST search option in the MalAvi database [10] (accessed on 27 March 2025). The population pairwise *F*_ST_ was used to test genetic differentiation of *Leucocytozoon* in different populations and black fly species. To test the hypothesis that *Leucocytozoon* lineages in black flies from different populations differed genetically, cyt b sequences of these protozoa from the same black fly collection were treated as a population regardless of species. Similarly, to test the genetic differentiation between *Leucocytozoon* lineages in different black fly species, all cyt b sequences of these protozoa from the same black fly species, regardless of sampling location, were treated as the same population. The pairwise *F*_ST_ analysis was calculated in Arlequin ver 3.5 [29] with 1023 permutations for statistical tests. The Bonferroni correction was used to adjust the significance level for multiple tests. We examined whether *Leucocytozoon* lineage assemblages differed between sampling locations and between black fly species, using analysis of similarity (ANOSIM) [30]. The ANOSIM analysis was performed in PAST version 1.81 [31].

To determine the congruence between black fly hosts and *Leucocytozoon* phylogenies, we used two global fit methods: Procrustean Approach to Cophylogeny (PACo) [32] and ParaFit [33]. The ML trees inferred from COI haplotypes (650 bp) (Supplementary Materials S1) of representative black fly species (6 species) and cyt b (473 bp) *Leucocytozoon* lineages (*n* = 56) (Supplementary Materials S2) reported in black flies from Thailand [16,23], including those found in this study, were used as input trees. The ML trees of black fly species (Supplementary Materials S3) and *Leucocytozoon* lineages (Supplementary Materials S4) were inferred in IQ-TREE [34,35] web server version (http://iqtree.cibiv.univie.ac.at/) (accessed on 19 April 2025). The best-fit model for the ML trees of black fly species and *Leucocytozoon* lineages based on the Bayesian information criterion were GTR+F+G4 and K3Pu+F+R2, respectively. The ultrafast bootstrap method with 1000 replications was used to calculate branch support for black fly species and *Leucocytozoon* ML tree analyses.

For the PACo analysis, significant associations of parasite and vector phylogenies were determined using the global goodness of fit statistic (residual sum of squares in Procrustes superimposition or m2xy) based on 100,000 permutations. PACo analysis was implemented in R 4.5.0, using the ape ver. 5.8.1 [36] and vegan ver. 2.8-0 [37] packages. For the ParaFit analysis, both global and individual host-parasite links were tested based on statistical significance values estimated from 999 permutations with the Cailliez correction for negative eigenvalues. The ParaFit analysis was performed in the software package ape in R 4.5.0.

## 3. Results

### 3.1. Leucocytozoon Prevalence and Diversity

**Figure 2:**
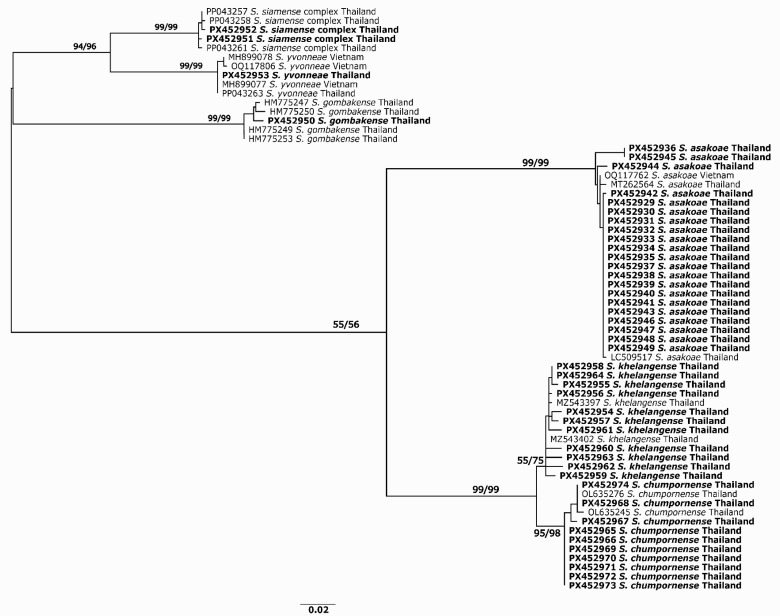
Maximum likelihood tree inferred from mitochondrial cytochrome oxidase I (COI) sequences from six species of black flies positive for *Leucocytozoon*. Bootstrap values based on 1000 pseudoreplicates for the ML and NJ analyses are above or near the branch. Bold indicates specimens in the present study.

We collected a total of 1081 adult black flies. Morphological identification supplemented with DNA barcoding revealed six species, all in the subgenus *Gomphostilbia* Enderlein, 1921: *Simulium asakoae*, *S. chumpornense*, *S. khelangense*, *S. siamense* complex, *S. gombakense* Takaoka and Davies, 1995 and *S. yvonneae* (Table 1, Figure 2). Two species, *S. khelangense* (*n* = 733) and *S. asakoae* (*n* = 256), represented the majority of specimens (Table 1). A total of 410 flies was used for molecular detection of *Leucocytozoon*, including representatives of *S. asakoae* (*n* = 150), *S. khelangense* (*n* = 220), *S. chumpornense* (*n* = 30), and all adults of the *S. siamense* complex (*n* = 6), *S. gombakense* (*n* = 2) and *S. yvonneae* (*n* = 2) (Table 1). In total, 46 individuals (11.2%) of six black fly species were positive for *Leucocytozoon* (Table 1), mostly *S. asakoae* (45.7%), *S. khelangense* (23.9%), and *S. chumpornense* (21.7%). Two individuals of the *S. siamense* complex and one each of *S. gombakense* and *S. yvonneae* also were positive. These latter three species are reported here for the first time as possible vectors of *Leucocytozoon*.

Twelve haplotypes (H1–H12) were identified among 46 cyt b sequences of *Leucocytozoon*. Comparisons of these cyt b haplotypes with those in GenBank indicated that most (8 of 12) are of an unidentified species (*Leucocytozoon* sp.) reported in chickens, with sequence similarity of >99%. One haplotype (H8) was identical to *Leucocytozoon schoutedeni* (accession no. DQ676823) in a chicken (*Gallus gallus* (L., 1758)) from Uganda. Haplotype H9 in *Simulium asakoae* was genetically highly similar (99%) to *Leucocytozoon* recorded in a raptor (accession no. MT281505). Three remaining haplotypes, two in the *S. siamense* complex (H10 and H11) and one in *S. yvonneae* (H12), were genetically distinct from all remaining *Leucocytozoon* in our study. The top blast hit for H10 was *Leucocytozoon* sp. (accession no. KX223864) in a Sandhill crane (*Grus canadensis* (L., 1758)) from the USA, with sequence similarity of 92%. H11 was most similar (92%) to *Leucocytozoon* sp. in the village weaver *Ploceus cucullatus* (Müller, 1776) (accession no. MT761629). H12 was genetically closest (92%) to *Leucocytozoon* sp. (accession no. LC782814) in wild birds from Japan.

Comparisons with haplotypes of *Leucocytozoon* in the MalAvi database [10] (accessed on 31 March 2025) showed that five haplotypes (H4–H8) were genetically identical with four existing lineages (GALLUS06, GALLUS17, GALLUS35, and GALLUS46). H6 and H7 matched 100% with GALLUS35 but were treated here as different haplotypes because they had single nucleotide differences in the final 20-bp of the cyt b sequences, which were longer than those in the MalAvi database. The remaining seven haplotypes, therefore, were new *Leucocytozoon* lineages.

### 3.2. Association of Leucocytozoon and Black Fly Species

**Figure 3:**
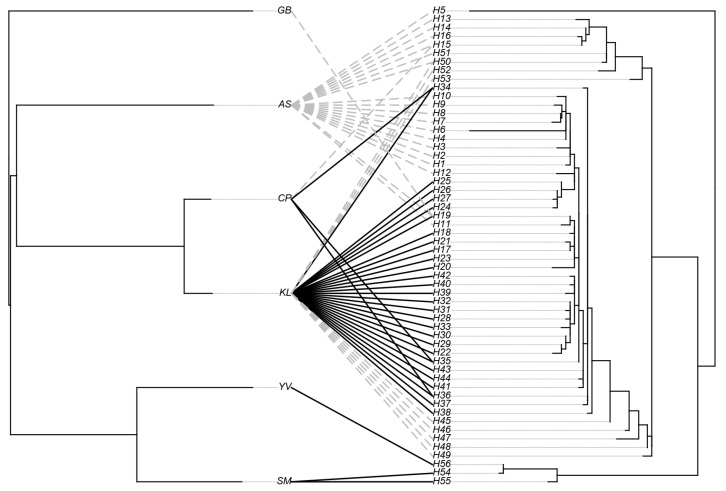
Tanglegram of black fly species and *Leucocytozoon* lineages. Connecting lines indicate black fly–*Leucocytozoon* associations; black lines denote significant co-phylogeny links between *Leucocytozoon* lineages and black flies (ParaFit tests, *p* < 0.05), dashed lines denote nonsignificant links (*p* > 0.05). AS, *S. asakoae*; CP, *S. chumpornense*; GB, *S. gombakense*; KL, *S. khelangense*; SM, *S. siamense* complex; YV, *S. yvonneae*.

Among 12 *Leucocytozoon* haplotypes, only two were shared by two or more black fly species (Table 2). H4 (*Leucocytozoon* sp.) and H8 (*L. schoutedeni*) were shared by *Simulium asakoae*, *S. khelangense*, and *S. gombakense* and by *S. asakoae* and *S. chumpornense*, respectively. *Leucocytozoon*–vector associations, based on Parafit tests, revealed significant co-phylogeny between black fly species and *Leucocytozoon* lineages (ParaFitGlobal value = 0.0171, *p* = 0.0024). Significant co-phylogeny was also supported by PACo analysis (m^2^xy = 0.475, *p* < 0.001; R^2^ = 0.525). Among 63 individual parasite–vector links, 34 exhibited significant *p*-values (<0.05) with ParaFit1 and ParaFit2 (Figure 3).

ANOSIM analysis revealed no significant difference between *Leucocytozoon* assemblages in different populations (R = −0.4375, *p* = 0.9728). However, *Leucocytozoon* assemblages in different black fly species differed significantly (R = 0.6406, *p* = 0.0284). The *F*_ST_ values indicated that only one pair of comparisons between *Leucocytozoon* from different populations differed significantly (Table 3). In contrast, all comparisons between *Leucocytozoon* lineages from different black fly species were significantly different except between the *Simulium siamense* complex and *S. khelangense* and between *S. asakoae* and *S. chumpornense* (Table 4).

## 4. Discussion

We report *Leucocytozoon* infections in *Simulium gombakense*, the *S. siamense* complex, and *S. yvonneae* for the first time. These black flies are in the subgenus *Gomphostilbia* and possess claws with a large basal tooth, an adaptation for feeding on birds [38]. Thus, the detection of avian haemosporidia in these species is not unexpected. The *Leucocytozoon* lineage in *S. gombakense*, which was also detected in *S. asakoae* and *S. khelangense*, corresponds to GALLUS17 in the MalAvi database [10], previously recorded from domestic chickens. Although the blood hosts of *S. gombakense* are unknown, the presence of this parasite suggests that *S. gombakense* feeds on chickens.

A *Leucocytozoon* lineage previously identified in raptors was detected in *Simulium asakoae*, providing the first evidence that this species probably feeds on raptors. *Leucocytozoon* lineages in the *S. siamense* complex and *S. yvonneae*, with only 92% sequence similarity to existing records from diverse hosts (e.g., Sandhill crane, village weaver, and unidentified wild birds from Japan), indicated that they are possible novel species [39]. These results highlight the need for further surveys of *Leucocytozoon* diversity in wild birds of Thailand, as well as blood-meal analysis of the *S. siamense* complex and *S. yvonneae* to identify the parasite sources.

The ParaFit test shows significant co-phylogeny between four black fly species and *Leucocytozoon* lineages. *Simulium khelangense* has the strongest signal, with 27 co-phylogenetic lineages, while its close relative *S. chumpornense* is significantly associated with three of these. However, both species also have multiple non-significant parasite associations, indicating they are generalist vectors. Similarly, *S. asakoae* transmits a broad diversity of *Leucocytozoon* lineages, consistent with its generalist feeding behavior.

Vector specificity in haemosporidian parasites could arise either as a by-product of host blood preference—where parasites are specific to avian hosts and vectors acquire them through blood feeding—or by direct vector–parasite coevolution. Such associations have been demonstrated across parasite genera (e.g., *Plasmodium*–Culicidae, *Haemoproteus*–Ceratopogonidae, and *Leucocytozoon*–Simuliidae) [40]. At broader taxonomic levels, however, both specialist and generalist relationships occur [41]. To date, at least 48 species of black flies have been incriminated as vectors or candidate vectors of *Leucocytozoon* [14,15,16,17,18,19], with most species exhibiting low specificity. Exceptions include *S. annulus*, which is specifically associated with lineage IGRYS1 due to its apparently restricted host associations with only certain groups of birds, notably cranes [6] and loons (family Gaviidae) [42].

Avian host associations of the black flies provide further context for understanding parasite–vector associations. *Simulium khelangense* and *S. chumpornense* primarily feed on chickens [43], but *S. khelangense* also feeds on turkeys [43] and owls [23]. *Simulium asakoae* feeds predominantly on chickens and occasionally on humans [43], and based on our study, it is also linked to raptors. These broad host ranges explain the high genetic diversity of *Leucocytozoon* lineages in these species. In contrast, the blood hosts of the *S. siamense* complex and *S. yvonneae* remain unknown, making it unclear whether their observed parasite associations result from host preferences or direct parasite–vector coevolution.

Although some of the black flies in our study are generalists, significant genetic structuring of *Leucocytozoon* lineages exists between coexisting black fly species, whereas differentiation among geographic populations is relatively low. An exception occurred in Ban Pla Ba, where lineages were predominantly restricted to *S. chumpornense*. Previous studies reported genetic differentiation of *Leucocytozoon* between *S. asakoae* and *S. khelangense* [23], with hypothesized temperature-driven coadaptation. However, our data do not support this explanation, as samples were collected under the same environmental conditions. Moreover, given that *S. asakoae*, *S. chumpornense*, and *S. khelangense* all feed primarily on chickens, genetic structuring is unlikely to result from host preference.

A more plausible explanation for genetic structuring of *Leucocytozoon* lineages between coexisting black flies is vector–parasite compatibility. Parasite development success differs significantly among vector–parasite combinations [44,45]. Vectors can evolve resistance mechanisms to limit parasite infections that reduce fitness, while parasites evolve immune-evasion strategies to complete their development [9]. Closely related black fly species are expected to share similar immune responses [46], favoring infection by genetically related *Leucocytozoon* lineages. Our findings support this idea: lineages associated with *S. chumpornense* are genetically similar to those in *S. khelangense*, with three of the four co-phylogenetic lineages shared between them.

## 5. Conclusions

Our study suggests that vector–parasite compatibility contributes to the observed associations between black fly species and *Leucocytozoon* lineages. Genetically similar *Leucocytozoon* lineages are either exclusively (e.g., *Simulium siamense* complex and *S. yvonneae*) or predominantly (e.g., *S. chumpornense* and *S. khelangense*) associated with closely related black flies. This pattern indicates that parasite adaptation to intrinsic vector traits, such as immunity [46,47] or microbiome composition [48], might play an important role in shaping vector specificity. Further research into these mechanisms is needed to elucidate the dynamics of *Leucocytozoon*–simuliid interactions. Our results for some species are based on limited sample sizes (*S. siamense* complex: *n* = 6; *S. gombakense*: *n* = 2; *S. yvonneae*: *n* = 2). Additional studies with larger samples are needed before firm conclusions can be drawn.

## Figures and Tables

**Table 1:** Black fly species, sampling locations, and number of female black flies collected (and used for molecular detection of *Leucocytozoon*) from Phu Ruea District, Loei Province, northeastern Thailand.

Species	Location (Code)	Coordinates	Elevation (m)	Collection Date	*n* Collected (*n* for Molecular Detection of *Leucocytozoon*, *n* Positive)
*S. asakoae*	Ban Nong Bua (617)	17.448202 N/101.343799 E	597	5 February 2023	21 (20, 1)
	Ban Pa Chan Tom (618)	17.457579 N/101.335994 E	590	5 February 2023	18 (18, 5)
	619			4 February 2023	46 (32, 8)
	Song Khon waterfall (620)	17.353900 N/101.404916 E	730	4 February 2023	80 (40, 6)
	Ban Pla Ba (626)	17.391766 N/101.371423 E	660	5 March 2022	91 (40, 1)
Total for *S. asakoae*					256 (150, 21)
*S. khelangense*	617				89 (30, 3)
	618				118 (30, 6)
	619				484 (100, 0)
	620				42 (30, 2)
Total for *S. khelangense*					733 (220, 11)
*S. siamense* complex	618				6 (6, 2)
*S. gombakense*	618				2 (2, 1)
*S. yvonneae*	618				2 (2, 1)
*S. chumpornense*	626				82 (30, 10)
Total					1081 (410, 46)

**Table 2:** Specimen counts for 12 *Leucocytozoon* haplotypes detected in six black fly species in northeastern Thailand. Haplotypes shared among two or more black fly species are in bold.

Haplotype	*S. asakoae*	*S. khelangense*	*S. chumpornense*	*S. siamense* Complex	*S. yvonneae*	*S. gombakense*
H1	17	0	0	0	0	0
H2	0	0	2	0	0	0
H3	1	0	0	0	0	0
**H4**	**1**	**11**	**0**	**0**	**0**	**1**
H5	0	0	1	0	0	0
H6	0	0	1	0	0	0
H7	0	0	3	0	0	0
**H8**	**1**	**0**	**3**	**0**	**0**	**0**
H9	1	0	0	0	0	0
H10	0	0	0	1	0	0
H11	0	0	0	1	0	0
H12	0	0	0	0	1	0
Total	21	11	10	2	1	1

**Table 3:** Population pairwise *F*_ST_ (below diagonal) and *p*-values (above diagonal) between *Leucocytozoon* lineages from five collections of black flies from northeastern Thailand, based on mitochondrial cyt b sequences.

Population	617	618	619	620	626
617		0.99099	0.39640	0.23423	0.17117
618	−0.12590		0.08108	0.33333	0.08108
619	0.03442	0.09536		0.16216	0.02703
620	0.11886	0.00169	0.10498		<0.00001
626	0.19268	0.15258	0.12053	**0.29618**	

Bold indicates statistical significance after Bonferroni correction of the significance level for multiple tests.

**Table 4:** Population pairwise *F*_ST_ (below diagonal) and *p*-values (above diagonal) between *Leucocytozoon* lineages in black flies from northeastern Thailand, based on mitochondrial cyt b sequences.

Black Fly Species	*S. asakoae*	*S. khelangense*	*S. siamense* Complex	*S. chumpornense*
*S. asakoae*		<0.00001	0.02703	0.00901
*S. khelangense*	**0.35587**		0.06306	<0.0001
*S. siamense* complex	**0.79411**	0.96885		<0.0001
*S. chumpornense*	0.23500	**0.41927**	**0.68726**	

Bold indicates statistical significance after Bonferroni correction of the significance level for multiple tests.

## Data Availability

The sequences have been deposited into the NCBI GenBank under the accession numbers PX452929-PX452974 and PX511765-PX511810. All other data and materials supporting this article are available from the corresponding author, P.P., upon request.

## Supplementary Materials

The following supporting information can be downloaded at: https://www.mdpi.com/article/10.3390/biology15010028/s1, S1: mitochondiral COI sequences of six black fly species; S2: mitochondrial cyt b sequences of 56 *Leucocytozoon* lineages; S3: maximum likelihood tree of six black fly species; S4: maximum likelihood tree of 56 *Leucocytozoon* lineages.
